# The dietary flavonoid isoliquiritigenin is a potent cytotoxin for human neuroblastoma cells

**DOI:** 10.1042/NS20180201

**Published:** 2019-01-30

**Authors:** Amnah M. Alshangiti, Katie L. Togher, Shane V. Hegarty, Aideen M. Sullivan, Gerard W. O’Keeffe

**Affiliations:** 1 Department of Anatomy and Neuroscience, University College Cork, Cork, Ireland; 2 Cork Neuroscience Centre, University College Cork, Cork, Ireland

**Keywords:** cell death, Molecular medicine, Neuroblastoma, small molecules

## Abstract

Neuroblastoma (NB) is the most common extracranial solid tumor of early childhood; it accounts for approximately 8–10% of all childhood cancers and is the most common cancer in children in the first year of life. Patients in the high-risk group have a poor prognosis, with relapses being common and often refractory to drug treatment in those that survive. Moreover, the drug treatment itself can lead to a range of long-term sequelae. Therefore, there is a critical need to identify new therapeutics for NB. Isoliquiritigenin (ISLQ) is a naturally-occurring, dietary chalcone-type flavonoid with a range of biological effects that depend on the cell type and context. ISLQ has potential as an anticancer agent. Here we show that ISLQ has potent cytotoxic effects on SK-N-BE(2) and IMR-32 human NB cells, which carry amplification of the *MYCN* gene, the main prognostic marker of poor survival in NB. ISLQ was found to increase cellular reactive oxygen species (ROS). The cytotoxic effect of ISLQ was blocked by small molecule inhibitors of oxidative stress-induced cell death, and by the antioxidant N-acetyl-l-cysteine (NAC). Combined treatment of either SK-N-B-E(2) or IMR-32 cells with ISLQ and the anticancer agent cisplatin resulted in loss of cell viability that was greater than that induced by cisplatin alone. This study provides proof-of-principle that ISLQ is a potent cytotoxin for MYCN-amplified human NB cells. This is an important first step in rationalizing the further study of ISLQ as a potential adjunct therapy for high-risk NB.

## Introduction

Neuroblastoma (NB) is the most common extracranial solid tumor of early childhood. It accounts for approximately 8–10% of all childhood cancers and is responsible for 15% of childhood deaths as a result of cancer [1]. NB arises from cells of sympathoadrenal lineage of the neural crest during development and often presents as tumors in the neck, chest, abdomen, and pelvis [2–4]. Age, stage, and tumor phenotypes are important prognostic factors in NB, and they guide treatment strategies [4]. In particular, amplification of the MYCN oncogene [5] and/or inactivation of p53 [6] are associated with increased disease risk and poorer outcomes. *MYCN* amplification is associated with rapid NB progression and poor prognosis [5,7], while p53 stabilization has been linked to multi-drug resistance in NB [8]. For those diagnosed with low- to intermediate-risk NB, the prognosis is relatively good, however many patients have high-risk metastatic disease that is resistant to multimodal therapy and that frequently relapses, resulting in a 5-year overall survival rate of approximately 50% [2]. Those patients that survive often have long-term sequelae as a result of toxicity associated with current chemotherapy regimens [9,10]. Therefore, there is a need for novel therapies for NB that have fewer off-target effects.

The study of naturally occurring pharmacological agents to prevent, inhibit, and delay carcinogenesis is a growing field of research, particularly in the area of cancer chemoprevention [11–13]. This strategy has been derived from epidemiological studies which suggest that diets rich in fruits and vegetables can reduce the risk of cancer [14,15]. Flavonoids have received significant attention in this regard, not only as preventative strategies, but also as potential chemotherapeutic agents [16,17]. In particular, isoliquiritigenin (ISLQ), a chalcone-derived flavonoid naturally found in liquorice and shallots [18], has been investigated for its anticancer properties due to its potent inhibition of cell proliferation and viability in a range of cancer cell types [19–29]. The anticancer effects of ISLQ on NB are yet to be studied, however ISLQ has recently been found to induce cytotoxicity in the pheochromocytoma (PC-12) cell line, which, like NB, has a neural crest origin [30], suggesting that ISLQ may have anticancer effects in NB cells.

The anticancer properties of ISLQ have been attributed to its ability to inhibit cell cycle progression, and to induce oxidative stress, apoptosis, autophagy, and/or necrosis. However, the precise mechanism of cytotoxicity is dependent on cell type and usually involves a combination of cell death pathways [22,24,26,31]. Understanding the mechanisms of cytotoxicity of ISLQ in NB cells will be important in determining its translational potential by improving knowledge of its potential off-target effects, its efficacy in tumor subtypes, and its usefulness as a possible combination therapy [32]. Here we sought to examine whether ISLQ treatment can exert anti-tumorigenic effects in MYCN-amplified NB cells, as the first step in rationalizing the study of ISLQ as a potential therapeutic agent for high-risk pediatric NB.

## Materials and methods

### Cell culture

Cells were purchased from the American Type Culture Collection (through Sigma). SK-N-BE(2) cells were maintained in Minimum Essential Medium (MEM) with 100 nM l-glutamine, 1% penicillin-streptomycin, 1% non-essential amino acid solution (100×), 1% F-12 Hams with 15% FBS. IMR-32 cells were maintained in MEM with 100 nM l-glutamine, 1% penicillin-streptomycin, 1% non-essential amino acid solution (100×) and 10% FBS. All reagents were from Sigma. Cells were cultured in a humidified atmosphere containing 5% CO_2_ at 37°C. After 2 days *in vitro*, when the cells had optimally adhered, cultures were treated with 5–100 µM ISLQ or a vehicle (equivalent percentage of DMSO; Sigma). For experiments involving inhibitors, cells were pre-treated with either: 50 µM Boc-D-FMK (Calbiochem), a cell-permeable, irreversible, broad-spectrum caspase inhibitor [37]; 10 µM necrostatin-1 (Nec-1; Bio-techne), an ATP-competitive, allosteric inhibitor of receptor-interacting protein kinase 1 (RIPK1) that blocks non-apoptotic cell death via inhibition of a specific cellular pathway, necroptosis, which leads to necrosis [39]; 3 µM IM-54 (Calbiochem), a small molecule inhibitor that selectively blocks oxidative stress-induced necrotic cell death; or 100 µM Bax-inhibiting peptide V5 (‘Bax inhibitor’; Calbiochem), a membrane-permeable pentapeptide inhibitor of Bax-mediated apoptosis [33]. As a control for the latter experiment, 100 µM of a corresponding cell-permeable control for the Bax-Inhibiting Peptide, that does not suppress Bax-mediated apoptosis (‘Bax control’; Calbiochem), was used. The antioxidant N-acetyl-l-cysteine (NAC; Sigma) was used at 1 mM. Cultures were pre-treated with the inhibitors for 1 or 2 h before treatment with 25 µM of ISLQ for 24 h. In other experiments, cultures were treated with 200 µM 6-hydroxydopamine (6-OHDA) for 4, 6, or 24 h or 10–100 µM cisplatin, a chemotherapeutic drug [34] (AdooQ Bioscience), for 24 h.

### Cell viability and death assays

For cell viability assays, cells were plated at a density of 1 × 10^5^ cells per well on 24-well plates. Phase contrast images were captured using an Olympus IX71 inverted microscope fitted with an Olympus DP70 camera. At each experimental end-point, MTT was added to the cells at a concentration of 1 mg/ml in culture medium for 4 h at 37°C. The culture medium was then removed and the formazon crystals were solubilized in DMSO. Absorbance was measured at 540 nm with a reference wavelength of 690 nm. Lactate dehydrogenase (LDH) activity was measured in 50 μl of cell culture medium of each treatment group using an LDH Activity Assay Kit (Sigma), according to the manufacturer’s instructions.

### Oxidative stress assessment

To analyze oxidative stress, cultures were treated with 25 µM of ISLQ for 24 h, after which the cell-permeable dye CellRox® (Thermo Fisher) was added at a final concentration of 5 µM, then cells were incubated for 30 min at 37°C. This dye exhibits bright green photostable fluorescence upon oxidation by reactive oxygen species (ROS) in living cells. The cells were washed in sterilized 10 mM PBS and four microscopic fields were randomly captured per well for each experiment, using an Olympus IX70 inverted microscope. The fluorescence intensity of individual cells was measured using ImageJ.

### RNA isolation, cDNA synthesis, and real-time PCR

For analysis of gene expression, cultures were treated with 5 or 25 µM of ISLQ for 24 h. The medium was removed and the cells washed in 10 mM PBS, before total RNA was extracted using TRIzol reagent as per the manufacturer’s instructions (Invitrogen). The quantity and quality of the mRNA was assessed using a NanoDrop 1000. Reverse transcription was carried out using the high capacity cDNA Reverse Transcription Kit (Applied Biosystems) under the following parameters: 25°C for 10 min; 37°C for 120 min; 85°C for 5 min; 4°C for 10 min. For real-time PCR, a reaction mix consisting of 0.5 µl of 20X Gene Expression Assays (*gapdh, bax, bcl2, caspase3*; TaqMan® Applied Biosystems), 0.5 µl of cDNA (20 ng/ml), 5 µl of TaqMan® Gene Expression Master Mix (Applied Biosystems) and 4 µl of RNase-free H_2_O (Applied Biosystems) was used. Each sample was run in duplicate under the following cycling parameters: 50°C for 2 min; 95°C for 10 min; 40 repetitions of 95°C for 15 s, and 60°C for 1 min. Gene expression levels were calculated using the 2^−δ*C*^_T_ method, normalizing to ACTB [35].

### Immunocytochemistry

For immunocytochemistry, cells were fixed in 4% paraformaldehyde in 10 mM PBS for 15 min. The paraformaldehyde was removed and the cells were washed three times for 5 min per wash in 10 mM PBS with 0.02% Triton (PBS-T). Non-specific binding was blocked by incubation in 5% BSA in PBS-T for 1 h at room temperature. The blocking solution was removed and primary antibody (Cell Signaling) to either cleaved caspase-3 or Bax, diluted 1:400 in 1% BSA in PBS-T, was added to the culture wells, followed by incubation overnight at 4°C. The primary antibody solution was removed, cells were washed in PBS-T, then incubated in Alexa Fluor 488–conjugated secondary antibody (1:1000; Invitrogen) diluted in 1% BSA in PBS-T, in the dark at room temperature for 2 h, then counterstained with DAPI. Five microscopic fields were randomly captured per well per experiment using an Olympus IX71 inverted microscope, and analyzed in a blinded fashion using ImageJ.

### Statistical analysis

A one-way ANOVA with *post-hoc* Tukey’s or Dunnett’s test was performed to measure any significant differences between groups. Results were expressed as mean ± S.E.M. and deemed significant when *P*<0.05.

## Results

### Effects of ISLQ treatment on cell viability in SK-N-BE(2) cultures

**Figure 1:**
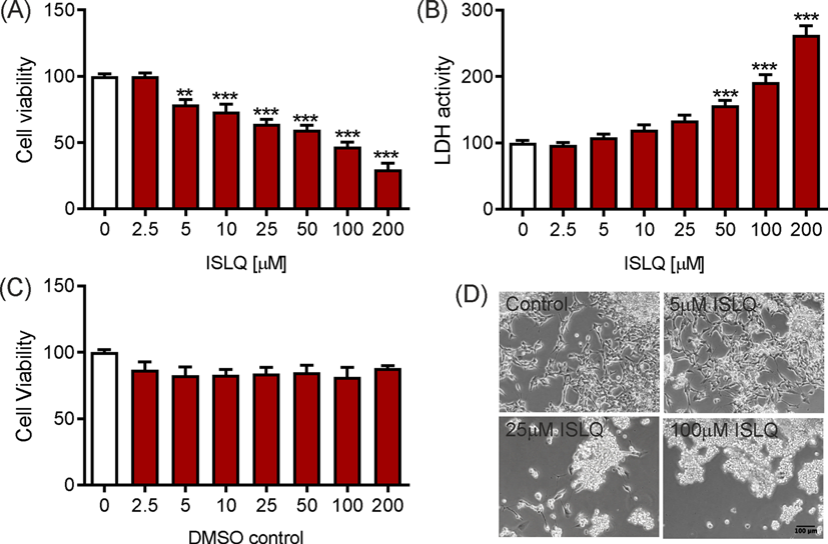
ISLQ induces cell death in SK-N-BE(2) NB cells Graphs of (**A**) MTT (*n*=9) and (**B**) LDH assays (*n*=9) on SK-N-BE(2) cells treated with ISLQ for 24 h. (**C**) Graph of an MTT assay (*n*=9) on SK-N-BE(2) cells treated with vehicle (DMSO) for 24 h. (**D**) Representative phase-contrast photomicrographs of ISLQ-treated cultures at 24 h. (**P<0.01, ***P<0.001 compared with control; ANOVA with *post-hoc* Tukey’s test). Scale bar = 100 µm. All data are mean ± S.E.M.

We first carried out a dose–response experiment on the effects of ISLQ treatment on cell viability in SK-N-BE(2) cultures. This cell line is MYCN-amplified [36] and therefore a suitable model of aggressive NB, given that MYCN amplification is the main prognostic marker of poor survival in NB [5]. Treatment with ISLQ at concentrations of 5 µM or greater resulted in a dose-dependent decrease (*P*<0.01) in cell viability in SK-N-BE(2), as measured by MTT assays (Figure 1A). This decrease in cell viability was not found in the corresponding control (DMSO vehicle only)-treated cultures (Figure 1C). To confirm these findings, LDH activity was examined in culture medium from ISLQ-treated SK-N-BE(2) cells. ISLQ treatment led to a significant increase (*P*<0.001) in LDH activity in SK-N-BE(2) cells, at concentrations of 50 µM or greater (Figure 1B). There were no changes in LDH activity in DMSO vehicle-treated cultures (data not shown). These results were confirmed by phase-contrast microscopy, which showed that ISLQ-treated cells displayed morphological features consistent with cell death (Figure 1D). Collectively, these data demonstrate that ISLQ treatment exerts concentration-dependent effects on viability in SK-N-BE(2) cells.

### The cytotoxic effects of ISLQ on SK-N-BE(2) cells are Bax- and caspase-independent

**Figure 2:**
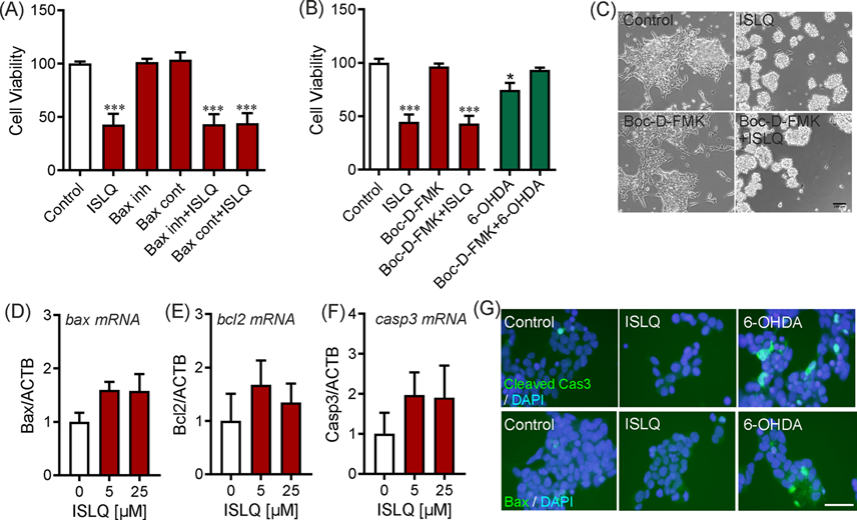
Inhibition of Bax or of caspase does not prevent ISLQ-induced death of SK-N-BE(2) cells (**A**,**B**) Graphs of MTT assays and (**C**) representative phase-contrast photomicrographs of SK-N-BE(2) cells treated with 25 µM ISLQ for 24 h with or without pre-treatment with (**A**) 100 μM of a Bax inhibitor peptide (‘Bax inh’) or of a control peptide (‘Bax cont’) (*n*=6) or (**B**) 50 µM of Boc-D-FMK (*n*=12). Treatment with 200 µM 6-OHDA (green bars) for 24 h was used as a positive control. Scale bar = 100 µm. (**D**–**F**) Graphs of real-time PCR data showing the relative expression of (**D**) *bax* mRNA, (**E**) *bcl2* mRNA, and (**F**) *caspase (casp)3* mRNA, normalized to that of β-actin (ACTB), in SK-N-BE(2) cells treated with non-cytotoxic (5 µM) and cytotoxic (25 µM) concentrations of ISLQ for 24 h (n=4). (**G**) Representative photomicrographs of SK-N-BE(2) cells treated with 25 µM ISLQ for 6 h, immunostained for cleaved caspase-3 or Bax. Treatment with 200 µM 6-OHDA was used as a positive control. (*P<0.05, ***P<0.001 compared with control; ANOVA with *post-hoc* Tukey’s test). Scale bar = 50 µm. All data are mean ± S.E.M.

Since the biological mechanisms mediating the effects of ISLQ are varied and cell-type-specific [18], we sought to determine the mode of cell death induced by ISLQ in SK-N-BE(2) cells. As ISLQ has been shown to increase the expression of Bax, decrease the expression of Bcl-2, and lead to caspase-dependent cell death in human carcinoma and hepatoma cells [37,38], we examined the involvement of Bax, Bcl-2, and caspases in ISLQ-induced cell death in SK-N-BE(2) cells. First, SK-N-BE(2) cells were pre-treated with 100 μM of a cell-permeable peptide (‘Bax inhibitor’) which is an effective inhibitor of Bax-mediated apoptosis, or with 100 μM of a control peptide (‘Bax control’), for 1 h prior to the addition of 25 µM ISLQ for 24 h. An MTT assay revealed that ISLQ treatment led to a significant decrease in cell viability in SK-N-BE(2) cells (*P*<0.001), which was not affected by pre-treatment with Bax inhibitor or Bax control (Figure 2A). Second, SK-N-BE(2) cells were pre-treated with 50 μM of a broad-spectrum small molecule caspase inhibitor (Boc-D-FMK) [39] for 2 h prior to the addition of 25 µM ISLQ for 24 h. An MTT assay showed that ISLQ-induced cell death in SK-N-BE(2) cells was not prevented by caspase inhibition (Figure 2B). Furthermore, treatment with 200 µM 6-OHDA, which has been reported to induce caspase-dependent cell death in this cell line [40], resulted in a significant decrease (*P*<0.05) in cell viability, which was prevented by pre-treatment with Boc-D-FMK (Figure 2B). Phase-contrast microscopy verified that pre-treatment with Boc-D-FMK did not prevent the cell death induced by ISLQ (Figure 2C). This lack of rescue of ISLQ-induced cell death by inhibition of either Bax or caspase was further supported by real-time PCR experiments, which showed that ISLQ treatment did not significantly alter the levels of expression of *bax* (Figure 2D), *bcl-2* (Figure 2E) or *caspase-3* (Figure 2F) mRNA in SN-N-BE(2) cells. In addition, and to further confirm these findings, we performed immunocytochemistry on SK-N-BE(2) cells using antibodies for Bax and for cleaved caspase-3. Following treatment of SK-N-BE(2) cultures for 4, 6, or 24 h with 200 µM 6-OHDA, significant numbers of cells stained positively for Bax and for cleaved caspase-3 (6 h time-point shown in Figure 2G). In contrast, there were no Bax- or cleaved caspase-3-positive cells in cultures treated with 25 µM ISLQ (Figure 2G). Collectively, these data suggest that the cytotoxic effects of ISLQ in SK-N-BE(2) cells are Bax- and caspase-independent, meaning that ISLQ induces cell death through other mechanisms in NB cells.

### The cytotoxic effects of ISLQ on SK-N-BE(2) cells are mediated by ROS-induced necrosis

**Figure 3:**
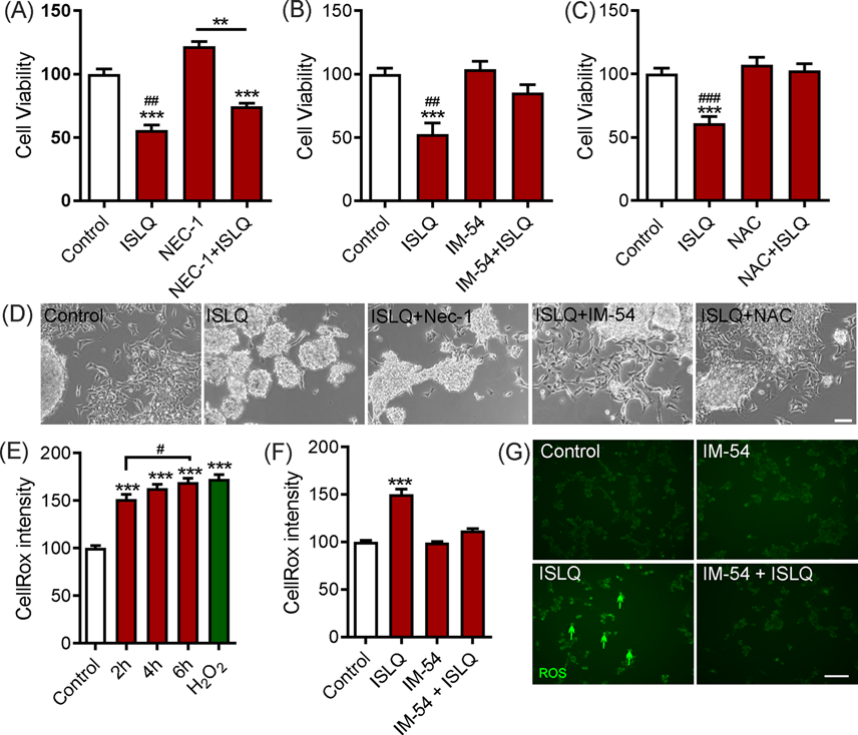
Inhibition of oxidative stress prevents ISLQ-induced death of SK-N-BE(2) cells (**A**–**C**) Graphs of MTT assays and (**D**) representative photomicrographs of SK-N-BE(2) cells treated with 25 µM ISLQ for 24 h with or without pre-treatment with (**A**) 10 μM Nec-1 necroptosis inhibitor) (*n*=9), (**B**) 3 μM IM-54 (inhibitor of oxidative stress-induced necrosis) (*n*=12) or (**C**) 1 mM NAC (antioxidant) (*n*=9). (**E**,**F**) Graphs and (**G**) representative photomicrographs showing relative fluorescence intensity of SK-N-BE(2) cells loaded with the CellRox fluorogenic probe that measures cellular oxidative stress (green). (**E**) Cells were treated with 25 μM ISLQ for 2, 4, or 6 h, or with 0.6 mM H_2_O_2_ for 30 min (*n*=80). (**F**) Cells were pre-treated with 3 μM IM-54 then cultured with or without 25 μM ISLQ for 24 h (*n*=240). (**P<0.01, ***P<0.001 compared with control or as indicated; ^#^*P*<0.05, ^##^*P*< 0.01, ^###^*P*<0.001 ISLQ compared with ISLQ + inhibitor; ANOVA with *post-hoc* Tukey’s test). Scale bar = 100 µm. All data are mean ± S.E.M.

Given that inhibition of either Bax or caspase did not block ISLQ-induced cell death, we next sought to determine if ISLQ induced cell death through necroptosis or necrosis. First, pre-treatment of SK-N-BE(2) cells for 2 h with Nec-1, a cell-permeable, potent, and selective inhibitor of necroptosis [39], was used. MTT assays showed that 25 µM ISLQ led to a significant decrease in SK-N-BE(2) cell viability (*P*<0.001) at 24 h, which was partially prevented by pre-treatment with 10 μM Nec-1 (Figure 3A,D). Pre-treatment for 1 h with 3 µM IM-54, a small molecule inhibitor that selectively blocks oxidative stress-induced necrotic cell death [41], fully blocked ISLQ-induced loss of cell viability in SK-N-BE(2) cells (*P*<0.001) (Figure 3B,D). To further investigate the involvement of oxidative stress in ISLQ-induced cell death, pre-treatment with the antioxidant NAC was used. An MTT assay showed that pre-treatment with 1 mM NAC fully prevented (*P*<0.001) ISLQ-induced cell death in SK-N-BE(2) cells (Figure 3C,D). To confirm the role of oxidative stress in ISLQ-induced cell death, the CellRox® fluorogenic probe was used to measure the cellular levels of ROS. SK-N-BE(2) cells were treated with ISLQ (25, 50, 100 μM) for 2, 4, and 6 h and then cultures were loaded with the CellRox® fluorogenic probe and fluorescence intensity was measured as a readout of cellular ROS. ISLQ treatment for 24 h was found to increase ROS levels, to a similar extent to those in cultures treated with a positive control (0.6 mM H_2_O_2_) (Figure 3E). The ISLQ-induced elevation of ROS levels was prevented by pre-treatment for 1 h with IM-54 (Figure 3F,G). These data suggest that ISLQ-induced cell death is mediated by ROS-mediated cell death.

### Effects of ISLQ treatment on cell viability in IMR-32 cultures

**Figure 4:**
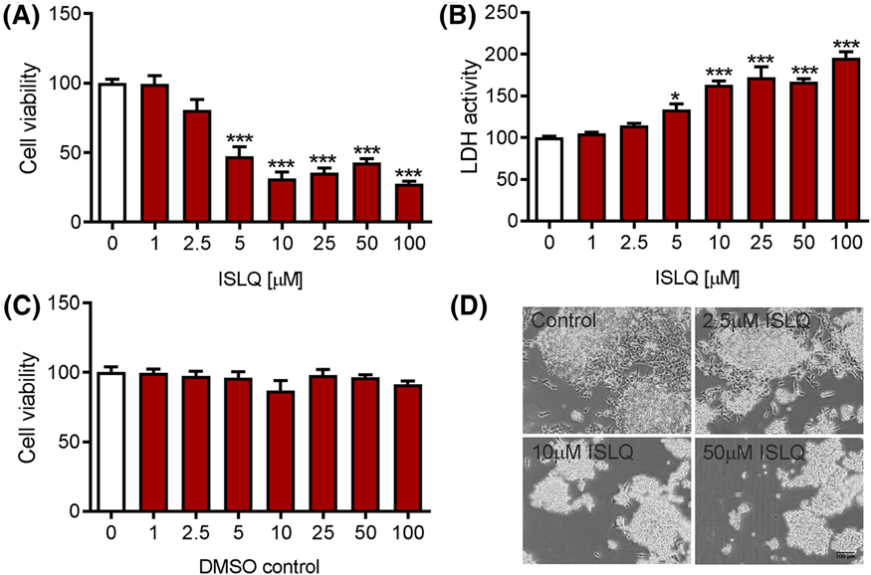
ISLQ induces cell death in IMR-32 NB cells Graphs of (**A**) MTT (*n*=9) and (**B**) LDH assays (*n*=9) on IMR-32 cells treated with ISLQ for 24 h. (**C**) Graph of an MTT assay on IMR-32 cells treated with vehicle (DMSO) for 24 h (*n*=9). (**D**) Representative phase-contrast photomicrographs of ISLQ-treated IMR-32 cells at 24 h. (*P<0.05, ***P<0.001 compared with control; ANOVA with *post-hoc* Tukey’s test). Scale bar = 100 µm. All data are mean ± S.E.M.

**Figure 5:**
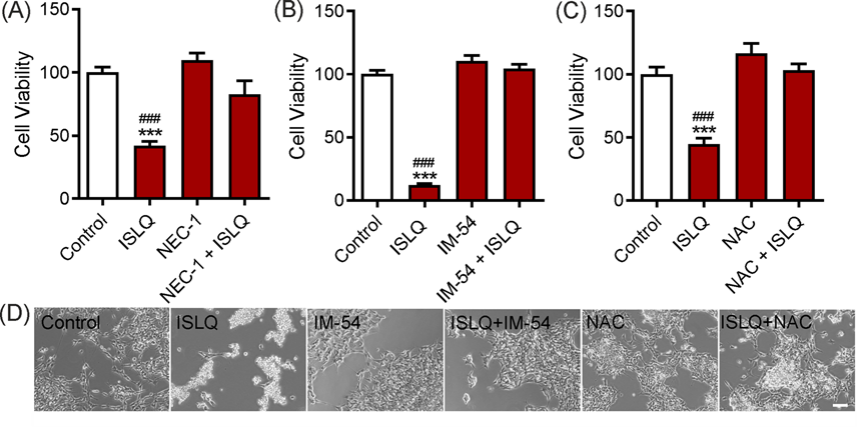
Inhibition of oxidative stress prevents ISLQ-induced cell death of IMR-32 cells (**A**–**C**) Graphs of MTT assay and (**D**) representative photomicrographs of IMR-32 cells treated with 25 µM ISLQ for 24 h with or without pre-treatment with (**A**) 10 μM Nec-1 (*n*=9), (**B**) 3 μM IM-54 (*n*=12), or (**C**) 1 mM of NAC (*n*=9). (***P<0.001 compared with control; ^###^*P*<0.001 ISLQ compared with ISLQ + inhibitor; ANOVA with *post-hoc* Tukey’s test). Scale bar = 100 µm. All data are mean ± S.E.M.

We next performed experiments to investigate the cytotoxic effects of ISLQ treatment in IMR-32 NB cells, another MYCN-amplified cell line. In agreement with our findings in SK-N-BE(2) cells, ISLQ treatment significantly reduced (*P*<0.001) IMR-32 cell viability at concentrations of 5 µM or greater, as measured using at MTT assay (Figure 4A,D), whereas there was no significant alteration in cell viability in cultures treated with DMSO vehicle (Figure 4C). These results were confirmed using an LDH assay, which showed that ISLQ treatment led to a significant increase (*P*<0.05) in LDH activity in IMR-32 cells, at concentrations of 5 µM or greater (Figure 4B). Moreover, in agreement with our data on SK-N-BE(2) cells, pre-treatment with Nec-1 (Figure 5A,D), IM-54 (Figure 5B,D), or NAC (Figure 5C,D) significantly prevented ISLQ-induced cell death in IMR-32 cells.

### Effects of combined treatment with ISLQ and cisplatin in MYC-amplified NB cells

**Figure 6:**
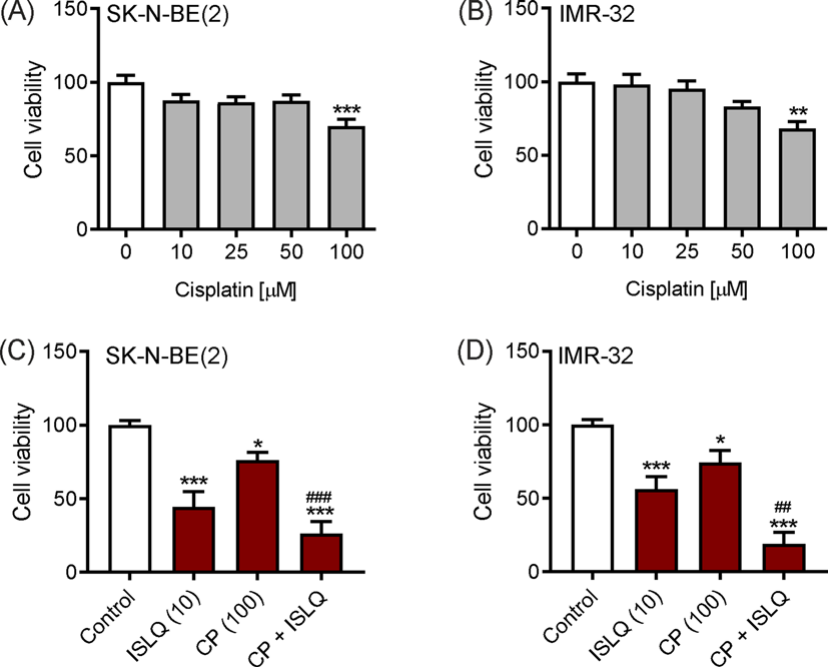
Effects of combined treatment with ISLQ and cisplatin on NB cells (**A**,**B**) Graphs of MTT assays on (**A**) SK-N-BE(2) cells (*n*=11) and (**B**) IMR-32 cells (*n*=10) treated with cisplatin (CP) for 24 h. (**C**,**D**) Graphs of MTT assays on (**C**) SK-N-BE(2) cells (*n*=6) and (**D**) IMR-32 cells (*n*=6) treated with 10 µM ISLQ for 24 h with or without 100 μM CP (concentrations shown in parentheses). (*P<0.05, **P<0.01, ***P<0.001 compared with control; ^###^*P*<0.001 compared with both ISLQ and CP in (**C**), ^##^*P*<0.01 compared with CP in (**D**); ANOVA with *post-hoc* Tukey’s test). Scale bar = 100 µm. All data are mean ± S.E.M.

To further explore the anticancer potential of ISLQ, we compared the cytotoxic effects of ISLQ treatment of both SK-N-B-E(2) and IMR-32 cells with those of a commonly used anticancer drug, cisplatin. First, we conducted a dose–response experiment using cisplatin alone and found that both cell lines were resistant to treatment with this agent at concentrations less than 100 µM (Figure 6A,B), while 100 µM cisplatin induced a significant loss of cell viability. Combined treatment of either SK-N-B-E(2) and IMR-32 cells with 10 µM ISLQ and 100 µM cisplatin for 24 h resulted in loss of cell viability that was significantly greater (*P*<0.001) than that induced by cisplatin alone (Figure 6C,D).

## Discussion

ISLQ is a naturally-occurring bioactive compound isolated from the roots of plants belonging to the liquorice family, with a range of biological effects that depend on the cell type and context [18]. Although the effects of ISLQ in a range of different cancers have been reported [23,26,42–48], to our knowledge there has been no study that has investigated the potential of ISLQ as a cytotoxic agent for NB. This is particularly important given the poor prognosis of NB, and the fact that many NB relapses are drug resistant, while current therapies can have a range of long-term sequelae [9,10]. Thus, the identification of new and/or adjunct therapeutic approaches for NB is essential. To address this, we used SK-N-BE(2) (p53 mutant) and IMR-32 cells to demonstrate that ISLQ exerts potent cytotoxic effects in both of these MYCN-amplified NB cell lines. SK-N-BE(2) cells, due to MYCN amplification and mutant p53, become highly resistant to apoptosis. One of the most important functions of p53 is its ability to activate apoptosis, and disruption of this process can promote tumor progression and chemoresistance [49]. We found that the cytotoxic effects of ISLQ in NB cells were mediated through oxidative stress-induced cell death, as evidenced by increases in cellular ROS levels, rather than through apoptosis or necroptosis. As a result, these data provide proof-of-principle that further study of ISLQ may be useful in developing new therapeutic approaches to high-risk NB.

In the present study, we found that ISLQ decreased viability of SK-N-BE(2) and IMR-32 cells when applied in the lower micromolar range, at concentrations of 5 μM or greater, for 24 h. This is similar to data reported in a recent study on the PC-12 neuronal cell line, which used a similar approach and found that ISLQ had a cytotoxic effect on these cells, with an IC_50_ of 17.8 µM [30]. Similarly, another study reported a cytotoxic effect of ISLQ on U87 glioma cells, with an IC_50_ of 6.3 µM, while it displayed minimal toxicity against normal brain cells [50]. A cytotoxic effect of ISLQ on SH-SY5Y cells has also been reported in a published abstract, which stated that treatment with 10–200 µM ISLQ for 24 h decreased cell viability [51]. This is fully consistent with our data on IMR-32 and SK-N-BE(2) NB cells, where ISLQ treatment for 24 h significantly reduced cell viability at concentrations of 5 µM or greater.

The precise mechanism of ISLQ-induced cytotoxicity in cancer cells is dependent on cell type and usually involves a combination of cell death pathways, including oxidative stress, apoptosis, autophagy, and/or necrosis [22,24,26,31]. Given that previous studies had reported that ISLQ treatment of human bladder cancer T24 cells led to the increase in the expression of *bax* and *caspase 3* transcripts [26], we hypothesized that ISLQ may have a similar effect in NB cells. However, we found no change in the expression of these genes in SK-N-BE(2) cells after treatment with cytotoxic concentrations of ISLQ. In agreement with this, a published abstract reported no significant changes in caspase3/7 activation in SH-SY5Y cells after treatment with 10–200 µM ISLQ for 24 h [51]. This is fully consistent with our real-time PCR and immunocytochemistry results in SK-N-BE(2) cells, and is also further supported by our finding that treatment with a broad-spectrum caspase inhibitor or with a Bax inhibitor did not prevent ISLQ-induced cell death. Also in support of this, a previous report demonstrated that ISLQ induced cell death and caspase activation in U87 glioma cells [27]. However, in that study, while treatment of U87 glioma cells with the pan-caspase inhibitor Z-VAD-FMK blocked ISLQ-induced caspase activation, it did not eliminate ISLQ-induced cell death [27]. In agreement, we also report that ISLQ-induced cell death was not prevented by a broad-spectrum caspase inhibitor or by a Bax inhibitor, suggesting that, at least in SK-N-BE(2) cells, ISLQ-induced cell death is independent of both Bax and caspase.

Necrosis has been considered as an accidental and non-regulated cell death process. However, recent studies have shed light on a new concept of regulated necrosis called RIPK-dependent necrosis (or necroptosis). The most prominent characteristics of this type of cell death are an involvement of RIPK1 kinase activation (which can be assessed by monitoring RIPK1 phosphorylation) and suppression by several RIPK1 inhibitors, including Nec-1 [52]. Currently, the primary method for confirming necroptosis involves detection of the key molecules that control this process (i.e. RIP1, RIP3, and MLKL) [53,54]. RIP1/3 has been found to promote anti-metastatic outcomes by regulating oxidative stress to kill metastatic tumor cells [55,56]. In the present study, we found that ISLQ-induced death in SK-N-BE(2) and IMR-32 cells was partially prevented by pre-treatment with nec-1, an inhibitor of RIPK1. The fact that the inhibition was partial may be explained by the finding that some necrosis inducers can act by directly activating RIPK3 or MLKL, by-passing RIPK1 activation [57]. In addition, we found that cell death induced by ISLQ was fully blocked by pre-treatment with IM-54, an inhibitor of oxidative stress-induced cell death [41]. This is supported by the demonstration that ISLQ treatment increased cellular ROS, and that the antioxidant NAC prevented ISLQ-induced cell death, in both cell lines tested in our study. A high concentration (1 mM) of NAC was used, due to the need to compensate for depletion of the cellular antioxidant, glutathione [58]. An involvement of ROS in necroptosis has been reported [59]. Moreover, several studies have shown that necroptosis is accompanied by increased ROS production in human [60] and murine [61] cancer cell types. This supports elevation of oxidative stress as a potential mechanism underlying cell death triggered by necroptosis. These findings are supported by studies showing that ISLQ treatment induced redox imbalance and increased oxidative stress in HepG2 hepatocellular carcinoma cells [62] and in HeLa cells [63]. Moreover, dietary flavonoids, including ISLQ, have been shown to be metabolized to pro-oxidant radicals [64]. Similar findings have been reported in neuronal-like cells; for example, PC-12 cells treated with 10 or 20 µM ISLQ for 24 h display increases in intracellular ROS [30]. This is very similar to the present study, in which SK-N-BE(2) treated with 25 µM ISLQ for 24 h showed an increase in intracellular ROS. This elevation of ROS levels in SK-N-BE(2) cells peaked quite early after ISLQ treatment, that is, between 2 and 4 h. This suggests that oxidative stress plays a substantial role in the initiation of cell death induced by ISLQ. Elevated rates of ROS have been detected in almost all cancers, where they promote many aspects of tumor development and progression. However, tumor cells also express increased levels of antioxidant proteins which can detoxify ROS, suggesting that a delicate balance of intracellular ROS levels is required for cancer cell function. The dependence of tumor cells and cancer stem cells on their antioxidant capacity makes them vulnerable to agents that dampen antioxidant systems. There is a realistic prospect for treatments aimed at dramatically increasing intracellular ROS to kill cancer cells by decreasing their antioxidant capacity [65,66]. The advantage of such a strategy is that normal cells are not significantly affected, since they have lower basal ROS levels and therefore are less dependent on antioxidants. However, it is worth noting that there are numerous instances where antioxidant effects of ISLQ have been reported. For example, treatment of human arterial smooth muscle cells with 10 µM of ISLQ, which is similar to the concentration used in the present study, has been reported to reduce ROS levels, despite the fact that it also significantly decreased cell viability [67]. Additionally, a recent study that performed microarray analysis on ISLQ-treated PC-3 prostate cancer cells reported a significant enrichment of differentially-expressed genes involved in the gene ontology category ‘*response to oxidative stress’* [42]. These data suggest that ISLQ can have both pro- and anti-oxidant activities, depending on the cell type. In the present study, we show that ISLQ treatment increases cellular ROS, and that this is involved in mediating the cytotoxic effects of ISLQ on human NB cells. It is also worth noting that a recent study identified ISLQ as a natural mimetic of rapamycin [68], which is an FDA-approved mTOR inhibitor that has anti-carcinogenic effects in NB cells [69]. Treatment of NB cells with rapamycin for 24 h has been shown to increase cellular levels of ROS [70], which is consistent with the increases in cellular oxidative stress in MYCN-amplified SK-N-BE(2) cells induced by ISLQ treatment for 24 h, found in our study. It is also worth noting that MYCN amplification has recently been shown to increase ROS, and to confer sensitivity to an ROS-augmenting agent in NB cells [71]. Our present study is consistent with this, as it found that ISLQ had cytotoxic effects at concentrations of 5 μM or greater, on MYCN-IMR-32 and MYCN-amplified SK-N-BE(2) cells. This suggests that MCYN-amplified NB may be particularly sensitive to ISLQ-induced ROS.

The up-regulation of endogenous antioxidant systems by cancer cells as an adaptation to situations of oxidative stress can limit the effectiveness of many chemotherapy agents [72]. Furthermore, ROS generated by the administration of such agents can lead to the damage of healthy tissues, resulting in numerous deleterious side-effects. For example, oxidative stress mechanisms are known to be involved in the cardiotoxicity mediated by anthracycline [73], and in the nephrotoxicity triggered by platinum compounds [74]; these are long-term serious sequelae associated with the use of these commonly-used chemotherapy agents. Pediatric NB has been reported to be resistant to the chemotherapeutic agent, cisplatin [75]. In the present study, we found that both SK-N-BE(2) and IMR-32 NB cells lines were resistant to treatment with cisplatin at concentrations less than 100 µM. Furthermore, we report synergistic effects of combined treatment with ISLQ and cisplatin, at relatively low doses. The use of such low doses in combination could minimize the advent of deleterious side-effects on other healthy tissues, which may occur following treatment with higher doses of cisplatin.

The present study has shown potent cytotoxic effects of the dietary flavonoid, ISLQ, on MYCN-amplified SK-N-BE(2) and IMR-32 NB cell lines. One limitation of the current study that will be important to address in future investigations is the need to identify the proteins and molecular pathways that mediate the link between ISLQ treatment and oxidative stress. Given the aggressive nature of MYCN-amplified NB, and the fact that NB relapses can be refractory to treatment, these data are an important first step in rationalizing the further study of ISLQ as a potential therapeutic agent for high-risk pediatric NB. Additionally, the evidence to support combination therapy of ISLQ with cisplatin is an important finding, that may allow lower doses of these agents to be used to reduce the incidence of serious long-term sequalae.

## Abbreviations

6-OHDA: 6-hydroxydopamine
ISLQ: isoliquiritigenin
LDH: lactate dehydrogenase
MEM: minimum essential medium
NAC: N-acetyl-l-cysteine
NB: neuroblastoma
Nec-1: necrostatin-1
PC-12: pheochromocytoma cell line
RIPK: receptor-interacting protein kinase
ROS: reactive oxygen species

## References

[1] Modak S. and Cheung N.K. (2010) Neuroblastoma: therapeutic strategies for a clinical enigma. Cancer Treat. Rev. 36, 307–317 10.1016/j.ctrv.2010.02.006 20227189

[2] Matthay K.K., Maris J.M., Schleiermacher G., Nakagawara A., Mackall C.L., Diller L. et al. (2016) Neuroblastoma. Nat. Rev. Dis. Primers 2, 16078 10.1038/nrdp.2016.78 27830764

[3] Cheung N.K. and Dyer M.A. (2013) Neuroblastoma: developmental biology, cancer genomics and immunotherapy. Nat. Rev. Cancer 13, 397–411 10.1038/nrc3526 23702928 PMC4386662

[4] Maris J.M. (2010) Recent advances in neuroblastoma. N. Engl. J. Med. 362, 2202–2211 10.1056/NEJMra0804577 20558371 PMC3306838

[5] Huang A. and Weiss W.A. (2013) Neuroblastoma and MYCN. Cold Spring Harb. Perspect. Med. 3, a014415 10.1101/cshperspect.a014415 24086065 PMC3784814

[6] Cattelani S., Ferrari-Amorotti G., Galavotti S., Defferrari R., Tanno B., Cialfi S. et al. (2012) The p53 Codon 72 Pro/Pro genotype identifies poor-prognosis neuroblastoma patients: correlation with reduced apoptosis and enhanced senescence by the p53-72P isoform. Neoplasia 14, 634–643 10.1593/neo.12594 22904680 PMC3421959

[7] Campbell K., Gastier-Foster J.M., Mann M., Naranjo A.H., Van Ryn C., Bagatell R. et al. (2017) Association of MYCN copy number with clinical features, tumor biology, and outcomes in neuroblastoma: a report from the Children’s Oncology Group. Cancer 123, 4224–4235 10.1002/cncr.30873 28696504 PMC5650521

[8] Xue C., Haber M., Flemming C., Marshall G.M., Lock R.B., MacKenzie K.L. et al. (2007) p53 determines multidrug sensitivity of childhood neuroblastoma. Cancer Res. 67, 10351–10360 10.1158/0008-5472.CAN-06-4345 17974978

[9] Perwein T., Lackner H., Sovinz P., Benesch M., Schmidt S., Schwinger W. et al. (2011) Survival and late effects in children with stage 4 neuroblastoma. Pediatr. Blood Cancer 57, 629–635 10.1002/pbc.23036 21319289

[10] Ducassou A., Gambart M., Munzer C., Padovani L., Carrie C., Haas-Kogan D. et al. (2015) Long-term side effects of radiotherapy for pediatric localized neuroblastoma: results from clinical trials NB90 and NB94. Strahlenther. Onkol. 191, 604–612 10.1007/s00066-015-0837-z 25896312

[11] Benetou V., Lagiou A. and Lagiou P. (2015) Chemoprevention of cancer: current evidence and future prospects. F1000Res. 4, 10.12688/f1000research.6684.1 27006756 PMC4797967

[12] Steward W.P. and Brown K. (2013) Cancer chemoprevention: a rapidly evolving field. Br. J. Cancer 109, 1–7 10.1038/bjc.2013.280 23736035 PMC3708589

[13] Landis-Piwowar K.R. and Iyer N.R. (2014) Cancer chemoprevention: current state of the art. Cancer Growth Metastasis 7, 19–25 10.4137/CGM.S11288 24987270 PMC4064948

[14] Bradbury K.E., Appleby P.N. and Key T.J. (2014) Fruit, vegetable, and fiber intake in relation to cancer risk: findings from the European Prospective Investigation into Cancer and Nutrition (EPIC). Am. J. Clin. Nutr. 100, 394S–398S 10.3945/ajcn.113.071357 24920034

[15] Key T.J. (2011) Fruit and vegetables and cancer risk. Br. J. Cancer 104, 6–11 10.1038/sj.bjc.6606032 21119663 PMC3039795

[16] Batra P. and Sharma A.K. (2013) Anti-cancer potential of flavonoids: recent trends and future perspectives. 3 Biotech. 3, 439–459 10.1007/s13205-013-0117-5 28324424 PMC3824783

[17] Chahar M.K., Sharma N., Dobhal M.P. and Joshi Y.C. (2011) Flavonoids: a versatile source of anticancer drugs. Pharmacogn. Rev. 5, 1–12 10.4103/0973-7847.79093 22096313 PMC3210013

[18] Peng F., Du Q., Peng C., Wang N., Tang H., Xie X. et al. (2015) A review: the pharmacology of isoliquiritigenin. Phytother. Res. 29, 969–977 10.1002/ptr.5348 25907962

[19] Ii T., Satomi Y., Katoh D., Shimada J., Baba M., Okuyama T. et al. (2004) Induction of cell cycle arrest and p21(CIP1/WAF1) expression in human lung cancer cells by isoliquiritigenin. Cancer Lett. 207, 27–35 10.1016/j.canlet.2003.10.023 15050731

[20] Zhang X., Yeung E.D., Wang J., Panzhinskiy E.E., Tong C., Li W. et al. (2010) Isoliquiritigenin, a natural anti-oxidant, selectively inhibits the proliferation of prostate cancer cells. Clin. Exp. Pharmacol. Physiol. 37, 841–847 20456427 10.1111/j.1440-1681.2010.05395.x

[21] Kwon G.T., Cho H.J., Chung W.Y., Park K.K., Moon A. and Park J.H. (2009) Isoliquiritigenin inhibits migration and invasion of prostate cancer cells: possible mediation by decreased JNK/AP-1 signaling. J. Nutr. Biochem. 20, 663–676 10.1016/j.jnutbio.2008.06.005 18824345

[22] Jung J.I., Chung E., Seon M.R., Shin H.K., Kim E.J., Lim S.S. et al. (2006) Isoliquiritigenin (ISL) inhibits ErbB3 signaling in prostate cancer cells. Biofactors 28, 159–168 10.1002/biof.5520280302 17473376

[23] Chen H.Y., Huang T.C., Shieh T.M., Wu C.H., Lin L.C. and Hsia S.M. (2017) Isoliquiritigenin induces autophagy and inhibits ovarian cancer cell growth. Int. J. Mol. Sci. 18, E2025 10.3390/ijms18102025 28934130 PMC5666707

[24] Wu C.H., Chen H.Y., Wang C.W., Shieh T.M., Huang T.C., Lin L.C. et al. (2016) Isoliquiritigenin induces apoptosis and autophagy and inhibits endometrial cancer growth in mice. Oncotarget 7, 73432–73447 27708238 10.18632/oncotarget.12369 PMC5341989

[25] Kim D.C., Ramachandran S., Baek S.H., Kwon S.H., Kwon K.Y., Cha S.D. et al. (2008) Induction of growth inhibition and apoptosis in human uterine leiomyoma cells by isoliquiritigenin. Reprod. Sci. 15, 552–558 10.1177/1933719107312681 18487228

[26] Si L., Yang X., Yan X., Wang Y. and Zheng Q. (2017) Isoliquiritigenin induces apoptosis of human bladder cancer T24 cells via a cyclin-dependent kinase-independent mechanism. Oncol. Lett. 14, 241–249 10.3892/ol.2017.6159 28693160 PMC5494874

[27] Zhou G.S., Song L.J. and Yang B. (2013) Isoliquiritigenin inhibits proliferation and induces apoptosis of U87 human glioma cells *in vitro*. Mol. Med. Rep. 7, 531–536 10.3892/mmr.2012.1218 23229626

[28] Ma J., Fu N.Y., Pang D.B., Wu W.Y. and Xu A.L. (2001) Apoptosis induced by isoliquiritigenin in human gastric cancer MGC-803 cells. Planta Med. 67, 754–757 10.1055/s-2001-18361 11731922

[29] Hsu Y.L., Kuo P.L., Lin L.T. and Lin C.C. (2005) Isoliquiritigenin inhibits cell proliferation and induces apoptosis in human hepatoma cells. Planta Med. 71, 130–134 10.1055/s-2005-837779 15729620

[30] Yang L., Neale B.M., Liu L., Lee S.H., Wray N.R., Ji N. et al. (2013) Polygenic transmission and complex neuro developmental network for attention deficit hyperactivity disorder: genome-wide association study of both common and rare variants. Am. J. Med. Genet. 162B, 419–430 10.1002/ajmg.b.32169 23728934 PMC4321789

[31] Yang H.H., Zhang C., Lai S.H., Zeng C.C., Liu Y.J. and Wang X.Z. (2017) Isoliquiritigenin induces cytotoxicity in PC-12 cells *in vitro*. Appl. Biochem. Biotechnol. 183, 1173–1190 10.1007/s12010-017-2491-7 28488118

[32] Cattley R.C. and Radinsky R.R. (2004) Cancer therapeutics: understanding the mechanism of action. Toxicol. Pathol. 32, 116–121 10.1080/01926230490426507 15209411

[33] Yoshida T., Tomioka I., Nagahara T., Holyst T., Sawada M., Hayes P. et al. (2004) Bax-inhibiting peptide derived from mouse and rat Ku70. Biochem. Biophys. Res. Commun. 321, 961–966 10.1016/j.bbrc.2004.07.054 15358121

[34] Dasari S. and Tchounwou P.B. (2014) Cisplatin in cancer therapy: molecular mechanisms of action. Eur. J. Pharmacol. 740, 364–378 10.1016/j.ejphar.2014.07.025 25058905 PMC4146684

[35] Schmittgen T.D. and Livak K.J. (2008) Analyzing real-time PCR data by the comparative C(T) method. Nat. Protoc. 3, 1101–1108 10.1038/nprot.2008.73 18546601

[36] Veas-Perez de Tudela M., Delgado-Esteban M., Cuende J., Bolanos J.P. and Almeida A. (2010) Human neuroblastoma cells with MYCN amplification are selectively resistant to oxidative stress by transcriptionally up-regulating glutamate cysteine ligase. J. Neurochem. 113, 819–825 10.1111/j.1471-4159.2010.06648.x 20180881

[37] Hirchaud F., Hermetet F., Ablise M., Fauconnet S., Vuitton D.A., Pretet J.L. et al. (2013) Isoliquiritigenin induces caspase-dependent apoptosis via downregulation of HPV16 E6 expression in cervical cancer Ca Ski cells. Planta Med. 79, 1628–1635 10.1055/s-0033-1350956 24214831

[38] Hsu Y.L., Kuo P.L. and Lin C.C. (2005) Isoliquiritigenin induces apoptosis and cell cycle arrest through p53-dependent pathway in Hep G2 cells. Life Sci. 77, 279–292 10.1016/j.lfs.2004.09.047 15878356

[39] D’Mello S.R., Aglieco F., Roberts M.R., Borodezt K. and Haycock J.W. (1998) A DEVD-inhibited caspase other than CPP32 is involved in the commitment of cerebellar granule neurons to apoptosis induced by K+ deprivation. J. Neurochem. 70, 1809–1818 10.1046/j.1471-4159.1998.70051809.x 9572264

[40] Kim S.-Y., Woo M.-S., Park J.-S., Hyun J.-W., Kim Y.-S. and Kim H.-S. (2010) The neuroprotective role of tissue inhibitor of metalloproteinase-2 in MPP+- or 6-OHDA-treated SK-N-BE(2)C and SH-SY5Y human neuroblastoma cells. Neurosci. Lett. 468, 136–140 10.1016/j.neulet.2009.10.084 19883732

[41] Dodo K., Katoh M., Shimizu T., Takahashi M. and Sodeoka M. (2005) Inhibition of hydrogen peroxide-induced necrotic cell death with 3-amino-2-indolylmaleimide derivatives. Bioorg. Med. Chem. Lett. 15, 3114–3118 10.1016/j.bmcl.2005.04.016 15878276

[42] Zhang B., Lai Y., Li Y., Shu N., Wang Z., Wang Y. et al. (2018) Antineoplastic activity of isoliquiritigenin, a chalcone compound, in androgen-independent human prostate cancer cells linked to G2/M cell cycle arrest and cell apoptosis. Eur. J. Pharmacol. 821, 57–67 10.1016/j.ejphar.2017.12.053 29277717

[43] Peng F., Tang H., Liu P., Shen J., Guan X., Xie X. et al. (2017) Isoliquiritigenin modulates miR-374a/PTEN/Akt axis to suppress breast cancer tumorigenesis and metastasis. Sci. Rep. 7, 9022 10.1038/s41598-017-08422-y 28827662 PMC5567123

[44] Jung S.K., Lee M.H., Lim D.Y., Kim J.E., Singh P., Lee S.Y. et al. (2014) Isoliquiritigenin induces apoptosis and inhibits xenograft tumor growth of human lung cancer cells by targeting both wild type and L858R/T790M mutant EGFR. J. Biol. Chem. 289, 35839–35848 10.1074/jbc.M114.585513 25368326 PMC4276852

[45] Chen X., Zhang B., Yuan X., Yang F., Liu J., Zhao H. et al. (2012) Isoliquiritigenin-induced differentiation in mouse melanoma B16F0 cell line. Oxid. Med. Cell. Longev. 2012, 534934 10.1155/2012/534934 23304254 PMC3529869

[46] Lee Y.M., Lim D.Y., Choi H.J., Jung J.I., Chung W.Y. and Park J.H. (2009) Induction of cell cycle arrest in prostate cancer cells by the dietary compound isoliquiritigenin. J. Med. Food 12, 8–14 10.1089/jmf.2008.0039 19298190

[47] Lee C.K., Son S.H., Park K.K., Park J.H., Lim S.S. and Chung W.Y. (2008) Isoliquiritigenin inhibits tumor growth and protects the kidney and liver against chemotherapy-induced toxicity in a mouse xenograft model of colon carcinoma. J. Pharmacol. Sci. 106, 444–451 10.1254/jphs.FP0071498 18360095

[48] Yamamoto S., Aizu E., Jiang H., Nakadate T., Kiyoto I., Wang J.C. et al. (1991) The potent anti-tumor-promoting agent isoliquiritigenin. Carcinogenesis 12, 317–323 10.1093/carcin/12.2.317 1899810

[49] Fridman J.S. and Lowe S.W. (2003) Control of apoptosis by p53. Oncogene 22, 9030 10.1038/sj.onc.1207116 14663481

[50] Zhao S., Chang H., Ma P., Gao G., Jin C., Zhao X. et al. (2015) Inhibitory effect of DNA topoisomerase inhibitor isoliquiritigenin on the growth of glioma cells. Int. J. Clin. Exp. Pathol. 8, 12577–12582 26722447 PMC4680392

[51] Escobar S.J.M., Munoz L., Fong G., Winnischofer S.M.B., Dennis J.M., Rocha M.E.M. et al. (2016) The flavonoid isoliquiritigenin is toxic to neuroblastoma cells and promotes necroptosis. Free Radic. Biol. Med. 100, S121 10.1016/j.freeradbiomed.2016.10.314

[52] Galluzzi L., Vitale I., Abrams J.M., Alnemri E.S., Baehrecke E.H., Blagosklonny M.V. et al. (2012) Molecular definitions of cell death subroutines: recommendations of the Nomenclature Committee on Cell Death 2012. Cell Death Differ. 19, 107–120 10.1038/cdd.2011.96 21760595 PMC3252826

[53] Wang T., Jin Y., Yang W., Zhang L., Jin X., Liu X. et al. (2017) Necroptosis in cancer: an angel or a demon? Tumour Biol. 39, 1010428317711539 10.1177/1010428317711539 28651499

[54] Weber K., Roelandt R., Bruggeman I., Estornes Y. and Vandenabeele P. (2018) Nuclear RIPK3 and MLKL contribute to cytosolic necrosome formation and necroptosis. Commun. Biol. 1, 6 10.1038/s42003-017-0007-1 30271893 PMC6123744

[55] Philipp S., Sosna J. and Adam D. (2016) Cancer and necroptosis: friend or foe? Cell Mol. Life Sci. 73, 2183–2193 10.1007/s00018-016-2193-2 27048810 PMC11108265

[56] Cho Y.S., Challa S., Moquin D., Genga R., Ray T.D., Guildford M. et al. (2009) Phosphorylation-driven assembly of the RIP1-RIP3 complex regulates programmed necrosis and virus-induced inflammation. Cell 137, 1112–1123 10.1016/j.cell.2009.05.037 19524513 PMC2727676

[57] Dillon C.P., Weinlich R., Rodriguez D.A., Cripps J.G., Quarato G., Gurung P. et al. (2014) RIPK1 blocks early postnatal lethality mediated by caspase-8 and RIPK3. Cell 157, 1189–1202 10.1016/j.cell.2014.04.018 24813850 PMC4068710

[58] van Zandwijk N. (1995) N-Acetylcysteine (NAC) and glutathione (GSH): antioxidant and chemopreventive properties, with special reference to lung cancer. J. Cell. Biochem. 59, 24–32 10.1002/jcb.240590805 8538205

[59] Lin Y., Choksi S., Shen H.M., Yang Q.F., Hur G.M., Kim Y.S. et al. (2004) Tumor necrosis factor-induced nonapoptotic cell death requires receptor-interacting protein-mediated cellular reactive oxygen species accumulation. J. Biol. Chem. 279, 10822–10828 10.1074/jbc.M313141200 14701813

[60] Ma Y.-m., Peng Y.-m., Zhu Q.-h., Gao A.-h., Chao B., He Q.-j. et al. (2016) Novel CHOP activator LGH00168 induces necroptosis in A549 human lung cancer cells via ROS-mediated ER stress and NF-κB inhibition. Acta Pharmacol. Sin. 37, 1381–1390 10.1038/aps.2016.61 27264312 PMC5057234

[61] Wang J.S., Wu D., Huang D.Y. and Lin W.W. (2015) TAK1 inhibition-induced RIP1-dependent apoptosis in murine macrophages relies on constitutive TNF-alpha signaling and ROS production. J. Biomed. Sci. 22, 76 10.1186/s12929-015-0182-7 26381601 PMC4574455

[62] Sun C., Wang Z.H., Liu X.X., Yang L.N., Wang Y., Liu Y. et al. (2015) Disturbance of redox status enhances radiosensitivity of hepatocellular carcinoma. Am. J. Cancer Res. 5, 1368–1381 26101703 PMC4473316

[63] Yuan X., Zhang B., Gan L., Wang Z.H., Yu B.C., Liu L.L. et al. (2013) Involvement of the mitochondrion-dependent and the endoplasmic reticulum stress-signaling pathways in isoliquiritigenin-induced apoptosis of HeLa cell. Biomed. Environ. Sci. 26, 268–276 23534467 10.3967/0895-3988.2013.04.005

[64] Galati G., Sabzevari O., Wilson J.X. and O’Brien P.J. (2002) Prooxidant activity and cellular effects of the phenoxyl radicals of dietary flavonoids and other polyphenolics. Toxicology 177, 91–104 10.1016/S0300-483X(02)00198-1 12126798

[65] Storz P. (2005) Reactive oxygen species in tumor progression. Front. Biosci. 10, 1881–1896 10.2741/1667 15769673

[66] Liou G.-Y. and Storz P. (2010) Reactive oxygen species in cancer. Free Radic. Res. 44, 10.3109/10715761003667554 20370557 PMC3880197

[67] Chen T., Deng S. and Lin R. (2017) The inhibitory effect of Isoliquiritigenin on the proliferation of human arterial smooth muscle cell. BMC Pharmacol. Toxicol. 18, 57 10.1186/s40360-017-0165-2 28716056 PMC5512881

[68] Aliper A., Jellen L., Cortese F., Artemov A., Karpinsky-Semper D., Moskalev A. et al. (2017) Towards natural mimetics of metformin and rapamycin. Aging (Albany N.Y.) 9, 2245–2268 10.18632/aging.101319 29165314 PMC5723685

[69] Misawa A., Hosoi H., Tsuchiya K. and Sugimoto T. (2003) Rapamycin inhibits proliferation of human neuroblastoma cells without suppression of MycN. Int. J. Cancer 104, 233–237 10.1002/ijc.10914 12569580

[70] Huang H., Chen J., Lu H., Zhou M., Chai Z. and Hu Y. (2017) Two mTOR inhibitors, rapamycin and Torin 1, differentially regulate iron-induced generation of mitochondrial ROS. Biometals 30, 975–980 10.1007/s10534-017-0059-1 29063293

[71] Wang T., Liu L., Chen X., Shen Y., Lian G., Shah N. et al. (2018) MYCN drives glutaminolysis in neuroblastoma and confers sensitivity to an ROS augmenting agent. Cell Death Dis. 9, 220 10.1038/s41419-018-0295-5 29445162 PMC5833827

[72] Castaldo S.A., Freitas J.R., Conchinha N.V. and Madureira P.A. (2016) The tumorigenic roles of the cellular REDOX regulatory systems. Oxid. Med. Cell Longev. 2016, 8413032 10.1155/2016/8413032 26682014 PMC4670861

[73] Angsutararux P., Luanpitpong S. and Issaragrisil S. (2015) Chemotherapy-induced cardiotoxicity: overview of the roles of oxidative stress. Oxid. Med. Cell Longev. 2015, 795602 10.1155/2015/795602 26491536 PMC4602327

[74] Karasawa T. and Steyger P.S. (2015) An integrated view of cisplatin-induced nephrotoxicity and ototoxicity. Toxicol. Lett. 237, 219–227 10.1016/j.toxlet.2015.06.012 26101797 PMC4516600

[75] D’Aguanno S., D’Alessandro A., Pieroni L., Roveri A., Zaccarin M., Marzano V. et al. (2011) New insights into neuroblastoma cisplatin resistance: a comparative proteomic and meta-mining investigation. J. Proteome Res. 10, 416–428 10.1021/pr100457n 21128686

